# Resolution by Gainesville Medical Society

**Published:** 1890-10

**Authors:** A. C. Scott, R. C. Black

**Affiliations:** Sec’y.; Gainesville, Texas; Pres.; Gainesville, Texas


### RESOLUTION AS TO A STATE BOARD OF HEALTH.

GAINESVILLE, TEXAS, September 11, 1890.

WHEREAS, From time to time the physicians of the State of Texas have used every honorable endeavor, without avail, to secure an enactment creating and establishing a State Board of Health; and

*Whereas*, From the experience of other States, and other good reasons, we believe that the interests of our State and people would be better protected, and served more efficiently and economically, and feeling that the importance of the creation of such Board has become so apparent and urgent that the question assuredly presents itself as a great public necessity; therefore be it

*Resolved*, By the Gainesville Medical Club that in the interests of the State and people, and for the public welfare generally, we unanimously favor and urge the creation of a State Board of

Health, and that we will unite with the medical profession of the State in unequivocally directing and calling the attention of the incoming administration and next Legislature to the vital and paramount importance of the question.

A. C. SCOTT, M. D., Sec'y. R. C. BLACK, M. D., Pres.

